# Laryngitis. Nitrate of Silver

**Published:** 1854-12

**Authors:** 


Laryngitis. Nitrate of Silver.—Dr. Ebert employs inhalations of nitrate of silver in substance with great benefit, in all inflammations of the laryngeal mucous membrane. He has employed the nitrate of silver also in solution, after the manner of Green, but has never been able to satisfy himself that the larynx was really entered. The mode in which the solid caustic is introduced is as follows: Three grains of the nitrate are mixed with one drachm of sugar; the powder is placed in a steel pen, which is itself firmly inserted in a quill open at both ends. The little apparatus is then put into the mouth, so that the end of the steel pen shall be on the root of the tongue; then the lips are closed round the quill, and the patient inspires forcibly. The first attempt is almost always a failure, and the nitrate is only tasted on the root of the tongue, but the patient soon learns to manage it very well; a little cough and irritation follow, but no great uneasiness. For young children this method does not answer, and a special apparatus must be used.—*Annalen des Berlin Char. Krankheiten.*

